# Rare breast and subcutaneous metastases from pancreatic neuroendocrine tumor: a case report

**DOI:** 10.1186/s12957-019-1662-0

**Published:** 2019-07-11

**Authors:** Dorotea Bosco, Salvatore Perrotti, Corrado Spatola, Giada Maria Vecchio, Rosalia Latino, Antonio Di Cataldo

**Affiliations:** 1 Department of General and Oncological Surgery, University of Catania, Via Santa Sofia 78, 95123 Catania, Italy; 2 Department of Oncology and Radiotherapy, University of Catania, Catania, Italy; 3 Department of Pathological Anatomy, University of Catania, Catania, Italy

**Keywords:** Pancreatic neuroendocrine tumors, Rare metastatic localization, Multiple metastases, Histological findings, Surgical treatment, Case report

## Abstract

**Background:**

Neuroendocrine tumors are a group of rare neoplasms, and the pancreatic neuroendocrine tumors (PNETs) represent only 1–2% of all pancreatic malignant tumors. The most common sites of these tumors include the gastrointestinal tract, lung, adrenal gland, and thyroid gland. Moreover, the most common sites of PNET metastases are the lymph nodes, liver, spleen, and bone.

**Case summary:**

A 40-year-old woman with pT3N1 PNET underwent surgical excision of the lesion (12 cm, at the level of the pancreatic body and tail). Postsurgical treatment included chemotherapy and radiation, both of which the patient showed a good tolerance for. After a 12-month disease-free interval, however, the patient reported the development of a lesion in her left breast and a small lesion in the left posterior region of her neck. The lesions were surgically excised, and the histological findings characterized both as pancreatic neuroendocrine metastatic poorly differentiated neoplasms (G3). A re-staging CT scan showed multiple metastases in the left axillary, clavicular, and latero-cervical lymph nodes, as well as diffuse osteolytic-osteoblastic bone metastases, almost mimicking the behavior of a primitive breast tumor.

**Conclusion:**

This case of breast and subcutaneous metastases from PNET should prompt awareness of potential metastatic lesions in unusual locations.

## Introduction

Neuroendocrine tumors represent a group of rare neoplasms, with an overall incidence of approximately 5.25/100000 [1]. The pancreatic neuroendocrine tumors (PNETs) represent only 1–2% of all pancreatic malignancy tumors, with incidence of 1–5 cases per million, mainly afflicting adults between the ages of 30 to 60 years; however, recent advances in imaging technologies and application of endoscopic ultrasound have led to an increase in the number of diagnosed PNETs [2–5]. Case reports in the literature have indicated that the most common sites of these tumors are the gastrointestinal tract, lung, adrenal gland, and thyroid gland [1, 6].

PNETs develop from the embryonic neural crest cells that later give rise to islet cell tissue and are classified as functioning or non-functioning depending on the presence of clinical manifestation secondary to the tumor cells’ increased hormonal secretion (i.e. insulin, gastrin vasoactive intestinal peptide, glucagon, and somatostatin) [3, 5, 6]. PNETs are also classified histologically as well differentiated, poorly differentiated, or mixed endocrine-exocrine subtypes [6]. The most common sites of PNETs’ metastases are reportedly the lymph nodes, liver, spleen, and bone [1].

We report herein the case of a woman with breast and subcutaneous PNETs’ metastases. Our paper is in line with the SCARE criteria [7].

## Case presentation

### Chief complaints

A 40-year-old woman presented with complaints of occasional abdominal pain, especially in the mesogastric region, vomiting, and alternating mucous diarrhea and constipation for about 2 years (since 2016).

### History of present illness

The patient reported that the symptoms had existed for about 2 years.

### History of past illness

The patient’s medical history was unremarkable.

### Physical examination

There were no remarkable findings on physical examination.

### Imaging examination

Abdominal ultrasound and computed tomography (CT) were performed and revealed a neoplasm (10 cm × 7 cm) with strong enhancement in the pancreatic body tail. We began to suspect PNET or sarcoma according to these imaging findings. The CT imaging also showed that, cranially, the tumor was in contact with the splenic artery but without signs of infiltration. In addition, an enlarged para-aortic lymph node (1.7 cm) was found below the left renal artery, near the left lower adrenal border. No intra/extrahepatic bile ducts’ dilation was observed.

### Cytological analysis

The patient underwent ultrasound-guided fine needle aspiration, and cytological analysis of the aspirate confirmed the PNET diagnosis.

### Surgical investigation and removal

**Fig. 1:**
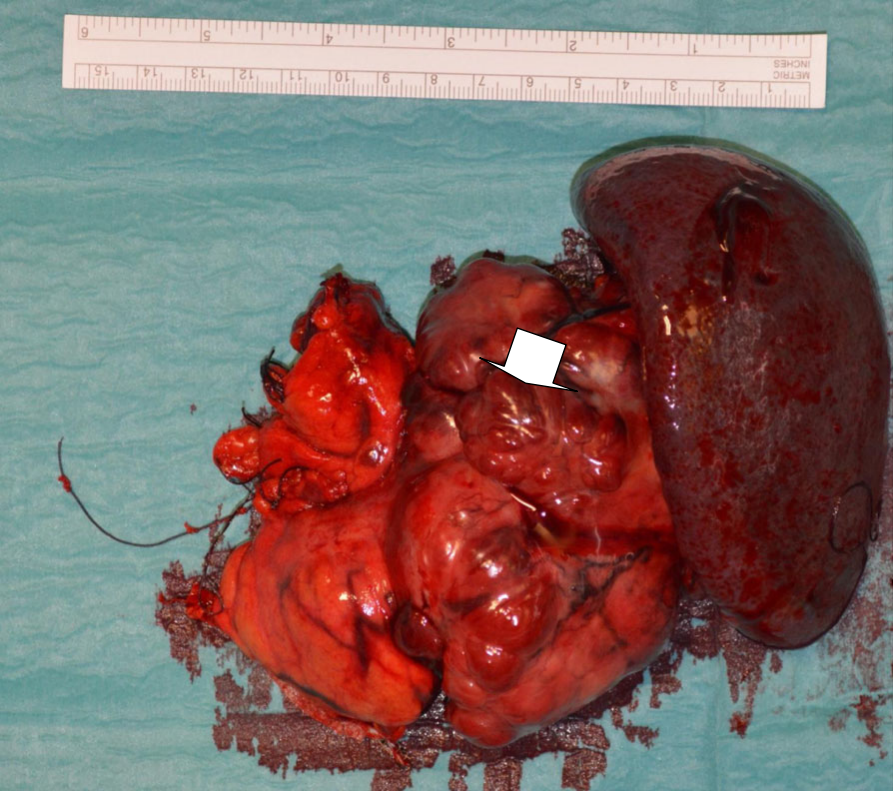
Intraoperative picture of the original PNET. The tumor is indicated by the arrow

Upon surgical investigation, a massive, hard lesion (12 cm) was found at the level of the pancreatic tail and determined to be causing a dislocation of the stomach (Fig. 1). The central region of the mass showed tenacious adhesion to the retroperitoneal wall, and a sample was sent for histological typing. Finally, a distal pancreatectomy with splenectomy was performed. No postoperative complications were observed, and the patient was discharged 8 days after the surgery. Histological analysis showed the spleen to be free of tumor cells but the retroperitoneum to be infiltrated by tumor cells (pT3N1). In addition, the lesion was confirmed to be a well-differentiated PNET (G2), with a poorly differentiated small component and perineural and vascular invasive growth (G3). Of the 6 lymph nodes excised, 1 was metastatic.

### Initial treatment of the PNET

After surgery, the patient underwent ^68^Gallium-DOTATOC positron emission tomography (commonly known as PET) imaging analysis, which produced no evidence of pathological uptake. Consequently, in October 2016, the adjuvant treatment was started, due to the high risk of local and distant relapse (high grade, lymphnodal metastases), consisting of combined concurrent radio chemotherapy, which was administered until January 2017. The radiotherapy was carried out by an intensity-modulated static step-and-shot technique to the surgical bed and locoregional lymphatic drainage, according to our institutional protocol [8]. A total dose of 6120 cGy, with daily fractionation of 180 cGy, was given to the planning target volume that had been defined according to the International Commission on Radiation Unit (commonly known as ICRU) Report 83 guidelines. The chemotherapy was carried out concomitant to radiation treatment, by means of an i.v. infusion of cisplatin (40 mg/mq weekly) and an oral administration of etoposide (100 mg on days 1–6 and 22–27).

The treatment regimen was well tolerated, with only a grade II gastro-intestinal toxicity (Common Toxicity Criteria of Adverse Event in the Clinic v4.2 recording system), which manifested nausea and vomiting. Subsequently, octreotide was administered s.c. every 28 days until October 2017, when a biochemical relapse was reported (chromogranin A (CgA) at 337 ng/mL), bringing an end to the 12-month disease-free interval. The patient also reported the development of a lesion in the left breast at this time.

### Physical examination of newly developed lesions

The patient’s breasts and nipples were grossly normal in shape and symmetric, without secretions. In the left axillary region, however, the skin was red with retraction. A palpable hard mass was found, which was adherent to the surrounding tissues. The patient also indicated the presence of a small subcutaneous lesion in the left posterior region of the neck.

### Surgical investigation and excision of the newly developed lesions

A quadrantectomy was performed on the upper outer portion of the left breast. Extemporaneous examination of the neck was carried out, followed by surgical excision of the lesion in the left posterior region.

## Multidisciplinary expert consultation

A pathologist was consulted to evaluate the excised lesions (Dr. Giada Maria Vecchio, Department of Pathological Anatomy, University of Catania, Catania, Italy). The surgical specimen comprised breast parenchyma with the dermis, measuring 6 cm × 3 cm × 4 cm.

Macroscopically, the specimen appeared as a bifocal, ill-defined solid mass, whitish in color, and hard in consistency, with a maximum diameter of 1.2 cm. This gross aspect was similar to that of a classic primitive breast neoplasm. The excised specimen also included a nuchal-type subcutaneous nodule, which was clinically interpreted as an inflamed dermic cyst that showed the same features of mammary nodules.

The histological examination of both neoplasms showed atypical polygonal cells with granular cytoplasm, round nuclei with fine “salt and pepper” chromatin, pseudoglandular and cord growth pattern, and marked desmoplastic reaction. The breast parenchyma around the tumor did not show any remarkable histological alterations and no evidence of hyperplasia or of ductal in situ carcinoma.

**Fig. 2:**
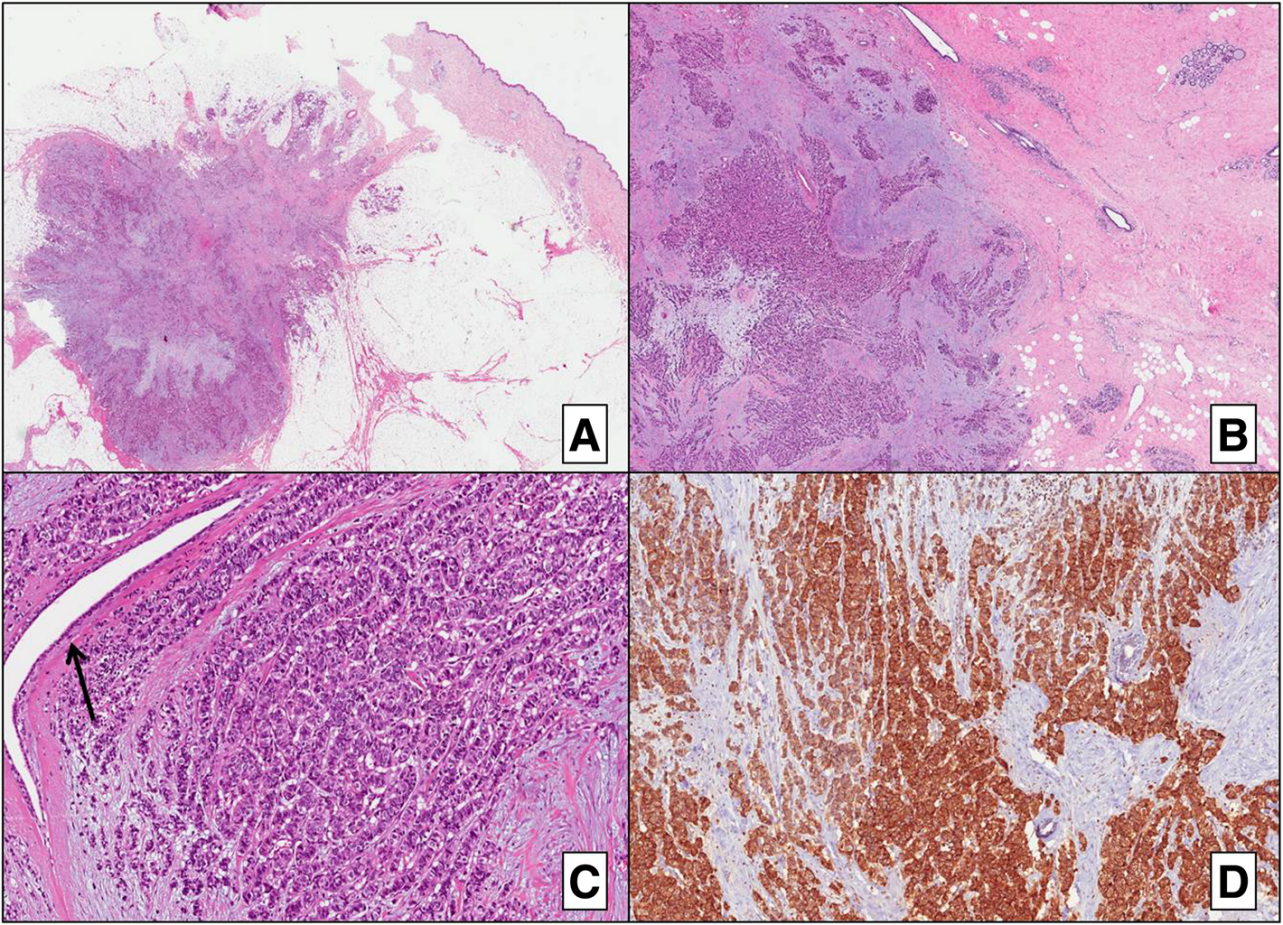
Immunohistochemistry results of the secondary lesions in the breast and subcutaneous tissue. **a** Low magnification showing mammary nodules with ill-defined and infiltrative margins near the epidermis (H&E; × 5). **b** Breast parenchyma around the tumor showing normal histologically findings. **c** High magnification showing the tumor with diffuse cord-like pattern associated with marked desmoplasia. An entrapped normal mammary duct is evident (arrow) (H&E; × 10). **d** Strong and diffuse immunoreactivity for chromogranin A. H&E hematoxylin and eosin

**Fig. 3:**
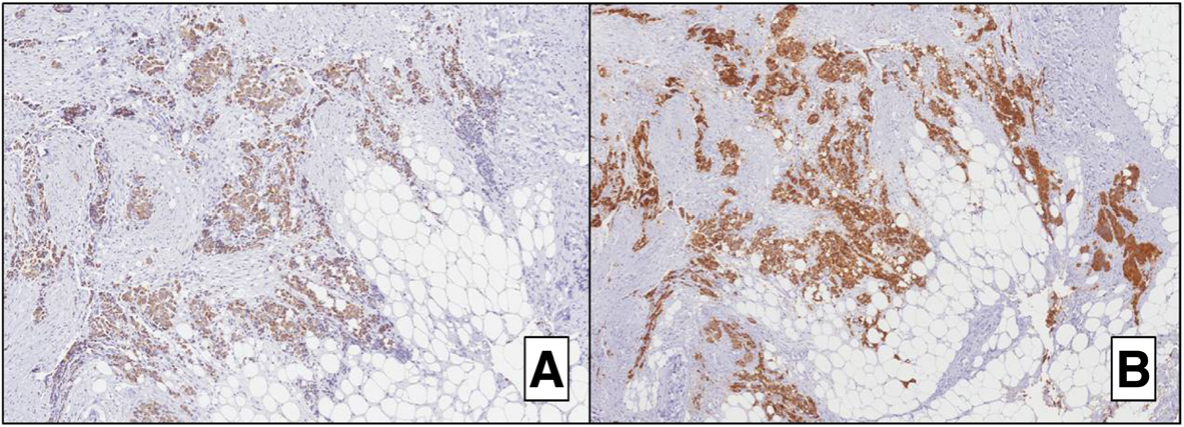
Strong and diffuse reactivity for CK19 (**a**) and synaptophysin (**b**)

The intraoperative histological exam suggested ductal invasive carcinoma of the breast. Immunohistochemical examination was carried out subsequently on formalin-fixed paraffin-embedded portions of the surgical specimen, using the labeled streptavidin-biotin peroxidase detection system and an automated immunostainer (Ventana, Tucson, AZ, USA). In consideration of the patient’s pathological anamnesis (prior diagnosis of poorly differentiated neuroendocrine carcinoma of the pancreas), a panel of numerous immunohistochemical markers, including classic breast ductal invasive carcinoma markers (i.e., estrogen and progesterone receptors, proliferative index/Ki67, and HER2) and additional neuroendocrine markers, was applied. The breast tumor cells did not show immunoreactivity for any of the ductal invasive carcinoma markers. However, both tissues showed strong positivity for CgA, synaptophysin, and CK19, indicating mammary and cutaneous metastases of the original PNET (Figs. 2 and 3).

## Final diagnosis

**Fig. 4:**
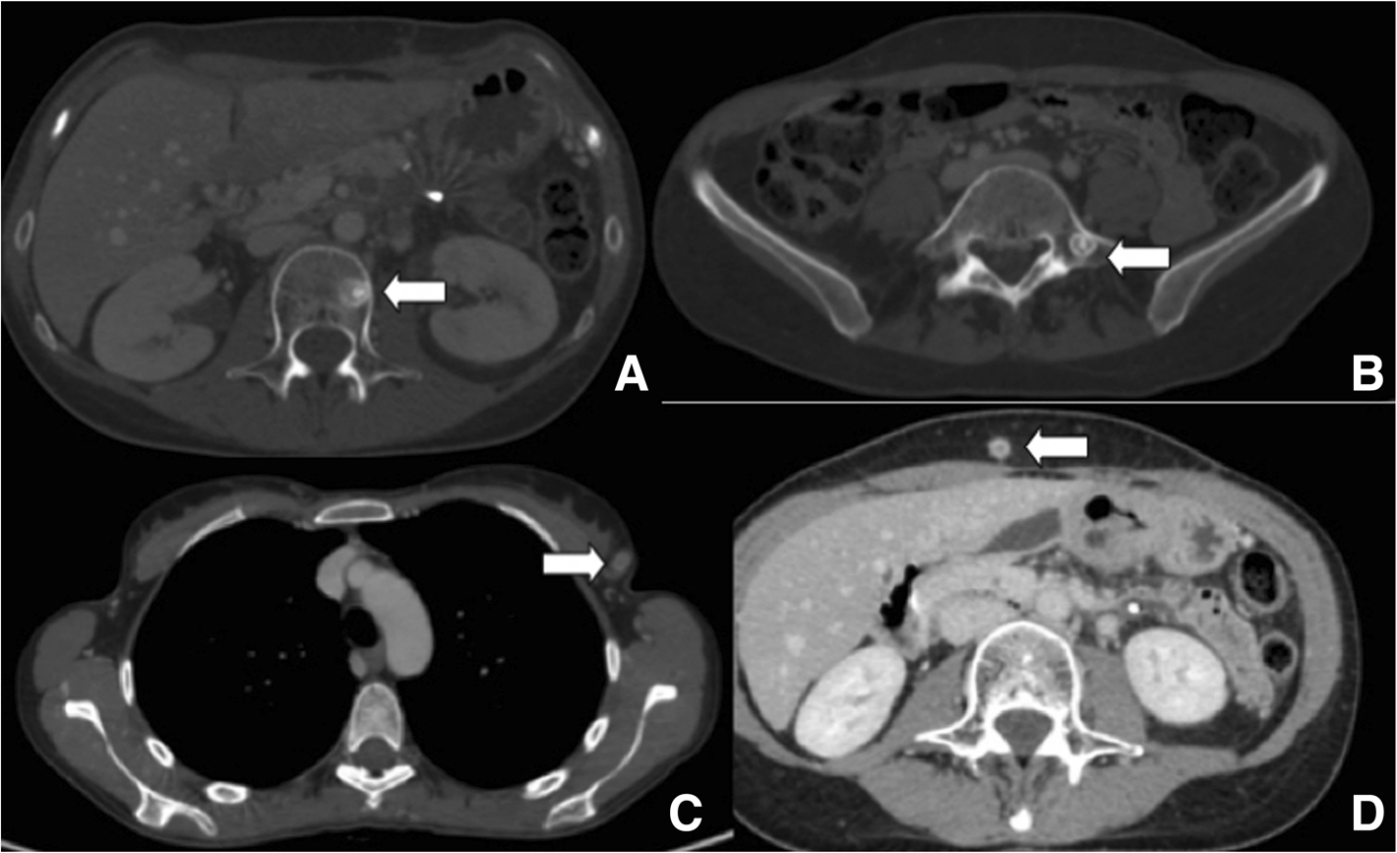
CT images at the time of systemic disease progression showing bone metastases in L3 (**a**) L5 (**b**) vertebra, left breast (**c**), and subcutaneous tissues of the abdominal wall (**d**)

The final diagnosis was metastatic poorly differentiated neoplasm for both lesions (G3), with a Ki67 expression distribution of 30%. Re-staging whole-body CT before surgery showed multiple metastases in the left axillary, clavicular, and latero-cervical lymph nodes and diffuse osteolytic-osteoblastic bone metastases, almost mimicking the behavior of a primitive breast tumor (Fig. 4). CT scan excluded the presence of further lesions in the parenchymatous organs (liver, lungs, brain). A second ^68^Gallium-DOTATOC PET was not performed due to a temporary unavailability of the PET center to synthesize the radiopharmaceutical drug.

## Treatment

After surgery, the patient underwent systemic chemotherapy treatment with a cisplatin 25 mg/mqon days 1–4 and etoposide 100 mg/mqon days 1–4, quoad 28 days, for 6 cycles until June 2018, and radiotherapy to address the bone lesions in the pelvis and the spine. At re-staging with whole-body CT scan, a disease progression was detected with the development of new multiple subcutaneous lesions and bone metastases. As a consequence of the systemic disease progression, a second-line treatment was activated by the use of octreotide s.c. quoad 28 days and everolimus. The latter is a targeted-therapy drug directed against the m-TOR (mammalian target of rapamycin) receptor; it is administered orally, at a dose of 10 mg/day without interruption.

## Outcome and follow-up

The patient’s treatment is ongoing at the time of writing of this report. At present, the patient shows a stable disease, both in bone metastasis to the pelvis, ribs, and spine and to the subcutaneous lesions. No grade III or greater side effects were detected, only a grade I–II oro-pharyngeal mucositis, treated with topical therapy and resolved. She continues systemic therapy with everolimus and octreotide for about 10 months, and disease stability has been confirmed by periodical whole-body CT scans.

## Discussion

PNETs derived from different neuroendocrine cells are a clinically rare and heterogeneous disease of the pancreas, accounting for 7% of all neuroendocrine tumors. The annual incidence of PNETs in the USA is estimated to range between 2 and 5 cases per 1,000,000 individuals, but, as stated above, the number of diagnosed cases has been increasing in recent years [2, 3]. Risk factors for PNETs include smoking, a high body mass index, and a positive family history which accounts for a variable percentage of patients with inherited syndromes, such as multiple endocrine neoplasia type 1 (known as MEN1) [9].

The common serological markers of PNETs, which develop from neural crest cells, are CgA and neuron-specific enolase, for each of which an abnormal increase often indicates the possibility of a neuroendocrine tumor [13]. The evolving neuroendocrine tumor classification systems have emphasized tumor grade and differentiation, in the assessment of the biologic aggressiveness of a neoplasm. Stratification of such tumors into low-, intermediate- and high-grade categories is crucial for predicting clinical behavior and guiding patient management [1]. The World Health Organization (commonly known as WHO) 2010, European Neuroendocrine Tumor Society, and American Joint Committee on Cancer systems are the most frequently used for classification and staging. The most widely accepted among the three, the WHO 2010 classification system, classifies PNETs into two categories (well differentiated and poorly differentiated tumors) based on mitotic count and Ki67 index, which has a prognostic and predictive value [10–14]. Yet, additional markers are needed to improve the prognostic classification of PNETs.

Recently, whole-exome and whole-genome sequencing studies have focused on identifying recurrent genetic alterations in primary PNETs. Among these alterations, the most commonly mutated genes are MEN1, DAXX (encoding the death domain-associated protein), and ATRX (encoding the alpha-thalassemia/mental retardation X-linked protein); the metastatic PNETs have additional mutations in the SETD2, ARID1A, and CDKN2A genes [15]. Up to 40–50% of patients with PNETs have a disseminated disease at initial diagnosis. The liver is a common site for metastasis from pancreatic neuroendocrine neoplasms, which can also occur in the course of tumor progression (occurring in 28.3–77% of cases) [9, 14, 16].

Metastatic PNETs have a worse 5-year overall survival rate (40–60%) than the metastatic intestinal neuroendocrine tumors (56–83%) [17]. Breast metastases from extramammary malignancies are uncommon in general (accounting for only 0.5–1.3% of cases) [18]. This rare occurrence of metastasis to the breast is suggested to be due to the presence of large areas of fibrous tissue that characteristically contain a relatively poor blood supply. No clear predisposing factors correlating with the development of breast metastasis have been identified, but hormones are considered to function as predisposing factors for several types of extramammary malignancies [18]. Cutaneous and subcutaneous metastasis from neuroendocrine tumors is also very rare, with less than 50 cases total reported in the literature [18].

Different imaging modalities are used to stage and localize PNETs. CT is considered the imaging technology of choice, as it is also routinely applied in the diagnosis and staging of cystic pancreatic tumors [19]. Magnetic resonance imaging is the second-line method of imaging for PNETs, and it has a greater sensitivity for detecting small tumors and liver metastasis than other modalities. Somatostatin receptor scintigraphy (also known as the OctreoScan) is often used when functional PNETs are suspected but tumors are not localized on cross-sectional images. PET with ^68^Gallium-DOTATATE has improved sensitivity. Endoscopic ultrasonography can also detect small tumors as well as lymph node involvement and vascular invasion; moreover, it can be used to assess fine needle aspirates or biopsies [11]. Due to the rarity of PNETs, there is limited evidence of the best management for such cases [8].

The current literature emphasizes that, today, patients with advanced non-resectable PNETs have several options for treatment, including combination therapy with synthetic analogs of the somatostatin receptor and modern molecular target drugs, such as those targeting the mTOR pathway (i.e. everolimus) or multipotential blocking agents against vascular endothelial growth factor and other receptor blocking agents, as well as peptide receptor radionuclide therapy or cytotoxic chemotherapy [16]. The development of molecular targeted agents has changed the landscape of treatment for PNETs [17]. According to the European Neuroendocrine Tumor Society (commonly known as ENETS) consensus guidelines, resection of metastases of grade 3 pancreatic neuroendocrine carcinoma is generally not recommended but may be considered in individual cases with isolated resectable metastases [2]. Active surveillance, especially for non-functioning tumors < 2 cm in size, should be considered although surgery remains the mainstay of treatment [9].

## Conclusion

PNETs are a heterogeneous group of tumors that a multidisciplinary team should be involved in determining the optimal treatment approach for according to the various factors connected to tumor stage and behavior. Our PNET case is distinctive for its metastases to breasts and subcutaneous tissue. As such, it highlights the importance of clinicians’ awareness of the possibility of a metastatic lesion when evaluating breast lesions in patients with primary tumor(s) in other organ(s), particularly PNETs.

## Experiences and lessons

- Cutaneous and subcutaneous metastasis from neuroendocrine tumors is very rare, with less than 50 reported cases in the literature.
- Immunohistochemical markers are very important to improve the prognostic classification of PNETs.
- PNETs are a heterogeneous group of tumors and their treatment approach should consider factors connected to the tumor stage and behavior, as assessed and discussed by a multidisciplinary team.

## Data Availability

All relevant data are within the manuscript.
